# Kidney-Related Screening Indicator Trends in Korea: 2010–2024 (eGFR, 2012–2024) and 2025–2028 Forecasts Using National Health Insurance Service Health Screening Data

**DOI:** 10.3390/medicina62071328

**Published:** 2026-07-09

**Authors:** Hyeran Jung, Minsun Jung

**Affiliations:** Department of Pathology, Yonsei University College of Medicine, Seoul 03722, Republic of Korea; phdgrace@yuhs.ac

**Keywords:** chronic kidney disease, estimated glomerular filtration rate, proteinuria, national health insurance service, health screening, epidemiology, Korea, forecasting, generalized linear model

## Abstract

*Background and Objectives*: Chronic kidney disease (CKD) risk classification depends on reduced estimated glomerular filtration rate (eGFR) and kidney-damage markers such as albuminuria or proteinuria. National screening statistics can identify population-level signals—referred to here as screening-based kidney risk indicators and abnormal screening prevalence—that serve as proxy indicators rather than confirmed CKD diagnoses, and require confirmatory testing when abnormal. *Materials and Methods*: This retrospective repeated cross-sectional ecological trend study analyzed National Health Insurance Service (NHIS) health-screening aggregate tables distributed through the Korean Statistical Information Service (KOSIS), representing individuals who underwent NHIS health screening rather than all insured individuals. Age- and region-stratified tables for eGFR, serum creatinine, urine protein, triglycerides, and hemoglobin were used. Non-eGFR indicators covered 2010–2024 and eGFR covered 2012–2024. Operational endpoints were eGFR < 60 mL/min/1.73 m^2^, serum creatinine ≥ 1.5 mg/dL, dipstick proteinuria ≥ 1+, triglycerides ≥ 150 mg/dL, and sex-specific anemia proxy; triglycerides and the anemia proxy were treated as associated metabolic/hematological indicators rather than kidney-specific endpoints. Crude national trends were supplemented with direct age-standardization (to the 2024 screening population) for eGFR, creatinine, proteinuria, and triglycerides, and with sex-stratified trends for all indicators. Binomial logit generalized linear models with calendar year as the predictor generated 2025–2028 forecasts; forecast reliability was assessed by temporal hold-out validation (fitting through 2020, predicting 2021–2024) and a recent-window (2018–2024) sensitivity analysis, and 95% bootstrap prediction intervals were derived by resampling model residuals and parameter uncertainty. Regional ecological correlations were assessed by Pearson and Spearman methods. *Results*: Dipstick proteinuria ≥ 1+ increased from 2.20% (age-standardized 2.34%) in 2012 to 3.71% in 2024 and was forecast to reach 4.64% (95% bootstrap prediction interval [PI], 4.50–4.83%) in 2028. eGFR < 60 decreased from 4.13% (age-standardized 5.20%) in 2012 to 3.43% in 2024, with a 2028 forecast of 3.06% (95% PI, 2.88–3.27%); the age-standardized decline was steeper than the crude decline, indicating the finding is not an artifact of population aging. Hold-out validation supported the eGFR, creatinine, and proteinuria forecasts (mean absolute error 0.05–0.14 percentage points) but showed poor fit for triglycerides and the anemia proxy, whose forecasts are reported as exploratory only. In 2024, eGFR < 60 reached 41.61% among persons aged ≥ 85 years. Jeonnam had the highest crude regional eGFR < 60 prevalence (5.09%), though regional comparisons could not be age-standardized. Regional eGFR < 60 was strongly correlated with elevated serum creatinine (Pearson r = 0.954, *p* < 0.001), reflecting their mathematical dependence rather than independent validation, and moderately correlated with the anemia proxy (Pearson r = 0.611, *p* = 0.009). *Conclusions*: NHIS/KOSIS screening data showed an age-standardization-robust upward proteinuria signal and persistent age-concentrated kidney function abnormalities. The findings support confirmatory testing after abnormal screening results and targeted kidney-risk follow-up, but they should not be interpreted as confirmed CKD incidence or prevalence.

## 1. Introduction

Chronic kidney disease (CKD) is a major public-health concern because reduced kidney function and kidney-damage markers are associated with cardiovascular disease, kidney failure, mortality, and health-system burden [1,2]. The global burden of CKD has been extensively documented, with estimates suggesting that approximately 9.1% of the world’s population is affected, and with the burden concentrated disproportionately among older adults and in regions with high rates of diabetes and hypertension [3,4]. More recent Global Burden of Disease estimates indicate that CKD has risen substantially in the ranking of causes of death and disability-adjusted life-years over the past three decades, a trend attributed to population aging, rising prevalence of diabetes and obesity, and improved survival with earlier, non-fatal stages of CKD that would previously have gone undetected [5]. CKD is typically classified using the Kidney Disease: Improving Global Outcomes (KDIGO) framework, which combines the estimated glomerular filtration rate (eGFR) category and albuminuria/proteinuria category into a composite risk grid; this classification requires both an estimate of excretory kidney function and a marker of structural kidney damage, each of which can be assessed through different, complementary laboratory tests [6,7].

South Korea provides a distinctive setting in which to study population-level kidney-risk indicators because of its near-universal National Health Insurance Service (NHIS) coverage and nationally mandated, employer- and government-linked biennial general health-screening program, which has operated at national scale since the early 2000s and includes routine serum creatinine, urine dipstick, and, more recently, eGFR reporting for the large majority of the insured adult population who elect to attend screening. Korea has also undergone rapid demographic aging over the past two decades, with the proportion of the population aged 65 years and older rising sharply, alongside a high and rising prevalence of type 2 diabetes and hypertension, the two leading causes of CKD worldwide [8]. These features make Korea’s national screening data a valuable, if imperfect, resource for tracking population-level kidney-risk signals over time, provided that the well-recognized limitations of aggregate, cross-sectional screening data—as opposed to individual-level, longitudinal clinical cohort data—are explicitly acknowledged throughout the analysis and interpretation.

Population health-screening programs can provide early signals of kidney risk, especially when screening abnormalities cluster by age group, sex, or region. However, screening results alone cannot establish a CKD diagnosis because clinical classification requires persistence of abnormal findings for at least three months and appropriate confirmatory testing [6,7]. The distinction between abnormal screening prevalence as proxy indicators rather than confirmed CKD diagnoses is essential for an appropriate interpretation of aggregate administrative data; a single abnormal screening result may reflect transient physiological variation, acute illness, medication effects, or laboratory measurement error rather than persistent kidney dysfunction, and only a minority of individuals with a single abnormal screening result will ultimately receive a confirmed CKD diagnosis after appropriate follow-up testing.

Reduced eGFR and proteinuria/albuminuria are the two indicators of most direct relevance to CKD risk classification. eGFR, most commonly estimated from serum creatinine together with age and sex using validated equations such as the Chronic Kidney Disease Epidemiology Collaboration (CKD-EPI) or Modification of Diet in Renal Disease (MDRD) equations, provides an indirect measure of excretory kidney function and declines progressively as functioning nephron mass is lost [6]. Proteinuria, detected here using semi-quantitative urine dipstick testing, reflects increased permeability of the glomerular filtration barrier to plasma proteins and is an independent marker of kidney damage and cardiovascular risk even when eGFR remains within the normal range [1]. Elevated serum creatinine and dipstick proteinuria have each individually been associated, in large population cohorts, with increased risk of all-cause mortality, cardiovascular events, and progression to kidney failure, with risk increasing steeply and continuously as eGFR falls and proteinuria rises [1,2]. Beyond these two primary indicators, elevated triglycerides and anemia are both well-documented, bidirectionally associated correlates of reduced kidney function: dyslipidemia arises partly from impaired lipoprotein clearance and altered lipase activity in CKD, while anemia arises predominantly from reduced renal erythropoietin production as functioning kidney tissue is lost, compounded by iron-handling abnormalities associated with chronic inflammation. Because these two indicators are widely available in national screening data and are clinically actionable in their own right (as cardiovascular and hematological risk factors, respectively), they are included in this analysis as associated metabolic and hematological screening indicators that commonly co-occur with, but are not equivalent or specific markers of, reduced kidney function; this conceptual distinction is described in full in Section 2.3.

Prior analyses have examined trends in eGFR, proteinuria, and related biomarkers in clinical cohorts and registry data, demonstrating strong associations between declining eGFR and adverse outcomes including end-stage renal disease and all-cause mortality [1,2,9]. Most of this evidence, however, derives from selected clinical cohorts, insurance claims among individuals already diagnosed with CKD or related comorbidities, or single-center registries, which may not represent the general screened population and cannot easily be used to monitor population-level temporal trends in kidney-risk indicators over a multi-year or multi-decade horizon. Nationally representative, repeated cross-sectional screening data offer a complementary source of population-level surveillance information, analogous to the role played by national health examination surveys in other countries, but population-level screening indicator trends among individuals undergoing NHIS health screening in Korea, stratified by age, sex, and region, and extended to short-term forecasting, have received comparatively little attention in the published literature [8,10]. In particular, we are not aware of prior published work that jointly (i) reports long-term (2010–2024) national trends across this specific panel of five screening indicators; (ii) distinguishes crude from age-standardized trends to separate true epidemiological change from population aging; (iii) generates validated, uncertainty-quantified short-term forecasts using a transparent statistical methodology appropriate for a short, annual, aggregate time series; and (iv) explicitly frames all endpoints as screening-based proxy indicators requiring confirmatory testing, rather than as directly interchangeable with confirmed CKD diagnoses.

This study used NHIS/KOSIS aggregate screening tables to evaluate national, age-specific, sex-specific, and regional kidney-related screening indicator trends in Korea through 2024 and to generate model-based forecasts for 2025–2028. The analysis focused on screening-based kidney risk indicators—proxy indicators rather than confirmed CKD diagnoses—including reduced eGFR, elevated serum creatinine, dipstick proteinuria, elevated triglycerides, and a sex-specific anemia proxy [11,12]. Specifically, this study aimed to (1) describe crude and age-standardized national annual trends in each indicator from 2010 (or 2012 for eGFR) through 2024; (2) characterize age-specific and sex-specific patterns in 2024; (3) describe regional variation and ecological correlations among indicators across 17 administrative regions in 2024; (4) generate and rigorously validate short-term (2025–2028) forecasts for each indicator, explicitly quantifying and communicating forecast uncertainty; and (5) interpret all findings within an explicit framework that distinguishes screening-based abnormal prevalence from confirmed CKD incidence or prevalence, in order to support appropriately cautious clinical and public-health interpretation of these national administrative data.

## 2. Materials and Methods

### 2.1. Study Design and Data Source

This was a retrospective repeated cross-sectional ecological trend study of aggregate health-screening statistics from the National Health Insurance Service (NHIS) [12] distributed through the Korean Statistical Information Service (KOSIS) [11]. The unit of observation was the calendar year, not the individual examinee; consequently, the design was repeated cross-sectionally and ecologically rather than longitudinally, and it could not track individual examinees across years. The study population comprised individuals who underwent the NHIS general health-screening examination in each calendar year, not all NHIS-insured individuals or the general Korean population; screening coverage among eligible insured adults is high (routinely cited at approximately 70–75% biennial participation nationally) but not complete, and non-participants may differ systematically from participants. The NHIS general health-screening program is offered biennially to insured adults aged ≥ 20 years (annually for specific occupational and age-based sub-groups); because screening is offered on a roughly two-year cycle, a given individual may or may not contribute an observation in any single calendar year, and the same individual may appear in the aggregate denominator of multiple years. Aggregate publicly released tables do not permit de-duplication of repeat examinees across years, which is acknowledged as a limitation in Section Limitations.

### 2.2. Data Extraction and Variable Construction

Age- and region-stratified distributions by sex for eGFR, serum creatinine, urine protein (dipstick), triglycerides, and hemoglobin were retrieved from the following publicly available KOSIS aggregate table series (all accessed on 15 May 2026 via https://kosis.kr): “Regional/Age-Specific Distribution of Estimated Glomerular Filtration Rate” (Sido-byeol/Yeonryeong-byeol Sinsagucheveogwayul Bunpohyeonhwang), “Regional/Age-Specific Distribution of Serum Creatinine” (Sido-byeol/Yeonryeong-byeol Hyeolcheongcreatinine Bunpohyeonhwang), “Regional/Age-Specific Distribution of Urine Protein” (Sido-byeol/Yeonryeong-byeol Yodanbaek Bunpohyeonhwang), and “Regional/Age-Specific Distribution of Triglycerides” (Sido-byeol/Yeonryeong-byeol Triglyceride Bunpohyeonhwang), each published by NHIS as part of the national health-screening statistical release. Non-eGFR indicators were available for 2010–2024; eGFR tables were available from 2012 onward because eGFR was not reported as a separate screening category before that year. Each table reports counts cross-tabulated by 5-year age band (≤19 to ≥85 years) and by sex, or by the 17 first-level administrative regions and sex, within pre-defined laboratory-value categories (for example, eGFR bands of <30, 30–39, 40–49, 50–59, 60–79, 80–99, 100–149, and ≥150 mL/min/1.73 m^2^). Operational thresholds used in this study (Section 2.3) were constructed by summing the relevant published categories; no re-binning of raw continuous values was required or possible because KOSIS releases only the pre-aggregated categorical counts. Cells suppressed by the data provider for small counts and any category with a non-numeric placeholder were treated as zero; this affected a small number of cells in the oldest age strata and did not materially affect national totals. Denominators differ across indicators because eGFR, creatinine, protein, and triglyceride panels are not always ordered together in the same screening visit, and because hemoglobin is drawn from the complete blood count component of the screening panel; each indicator’s denominator is therefore the number of examinees with a valid result for that specific test in that year, not the total number of screening participants. The tables do not report fasting status, assay platform, or whether laboratory methods or reference ranges changed over the study period; this is acknowledged as a limitation (Section Limitations). Age- and sex-specific counts in the source tables were used directly as published; no further aggregation, imputation, or recoding was performed by the authors beyond summing category counts into the operational endpoints defined in Section 2.3.

### 2.3. Operational Endpoints

Reduced eGFR was defined as <60 mL/min/1.73 m^2^ in accordance with KDIGO 2024 guidelines [6]. The KOSIS aggregate tables report pre-classified eGFR category counts as released by NHIS; the specific estimating equation (e.g., CKD-EPI 2009, CKD-EPI 2021, or MDRD) and whether it changed between 2012 and 2024 is not disclosed in the publicly released aggregate tables and could not be independently verified for this analysis. Any change in the estimating equation or in creatinine assay calibration over the study period could produce an apparent temporal change in reduced-eGFR prevalence that does not reflect a true change in kidney function; this is an important limitation of using pre-aggregated, equation-agnostic public statistics and is discussed further in Section Limitations. Elevated serum creatinine was defined as ≥1.5 mg/dL, a threshold commonly used in prior Korean NHIS-based screening reports as an approximate, assay-based marker of substantially reduced kidney function [1,2]; because a single absolute threshold does not account for age, sex, or muscle mass, this indicator will under-ascertain reduced kidney function in many older adults and in women, and is reported as a supplementary, more specific but less sensitive marker alongside eGFR rather than as a primary endpoint. Dipstick proteinuria was defined as ≥1+, following conventional semi-quantitative dipstick reporting used in the NHIS screening program [6,7]. Elevated triglycerides were defined as ≥150 mg/dL, the threshold used in the National Cholesterol Education Program/Adult Treatment Panel III and subsequent metabolic syndrome criteria [13]. The anemia proxy was sex-specific: hemoglobin < 13 g/dL for men and <12 g/dL for women, following World Health Organization anemia thresholds [6,7]. Reduced eGFR and dipstick proteinuria are the two indicators directly relevant to CKD risk classification under KDIGO criteria. Elevated triglycerides and the anemia proxy are not specific or equivalent markers of kidney disease: the triglyceride threshold reflects a cardiovascular and metabolic risk indicator that is frequently co-elevated with, but mechanistically distinct from, reduced kidney function, while the anemia thresholds applied here are general population anemia definitions rather than kidney-specific anemia criteria (which would additionally require the exclusion of iron deficiency, inflammatory, and other non-renal causes, information unavailable in these aggregate tables). These two indicators are therefore reported and interpreted throughout as associated metabolic and hematological screening indicators that commonly co-occur with reduced kidney function at the population level, rather than as kidney-screening endpoints in the same sense as eGFR and proteinuria; this distinction is now stated explicitly here and reiterated in Section 4.

### 2.4. Statistical Analysis

Annual crude prevalence was calculated as the abnormal count divided by the total examined count for each indicator and year. For the anemia proxy, male and female abnormal counts were calculated using sex-specific thresholds and then summed for total prevalence. National crude annual prevalence is reported alongside age-standardized annual prevalence (Section 2.5) so that temporal changes in the age composition of the screened population can be distinguished from changes in age-specific abnormality rates. Sex-stratified national annual prevalence and forecasts are additionally reported for all five indicators (Section 3.1) because abnormality rates and trends differed materially by sex. Region-level ecological correlations in 2024 were assessed across 17 administrative regions using Pearson and Spearman correlations, with exact 95% confidence intervals calculated for each correlation coefficient using Fisher’s z-transformation; given that six pairwise correlations were tested, results are interpreted with attention to multiple comparisons rather than as a family of independent hypothesis tests. Two-sided *p* values < 0.05 were considered statistically significant, except where noted otherwise. All statistical analyses were performed in R version 4.3 (R Foundation for Statistical Computing, Vienna, Austria).

### 2.5. Age Standardization

Because the screened population’s age structure is likely to have shifted over the 13–15-year study window and reduced eGFR, elevated creatinine, and the anemia proxy are all strongly age-dependent (Section 3.2), crude national annual prevalence for eGFR, creatinine, proteinuria, and triglycerides was additionally directly age-standardized to a fixed reference population, defined as the observed 2024 national screening population’s age distribution (16 five-year age bands from ≤19 to ≥85 years), using sex-combined age-specific counts from the corresponding KOSIS age-stratified tables. For each year, the standardized rate was calculated as the sum, over the 16 reference age bands, of the year- and age-band-specific crude abnormality rate multiplied by the corresponding 2024 reference-population weight. Age-standardized trends could not be computed for the anemia proxy because an age-stratified hemoglobin table was not available at the time of this analysis; this is noted as a limitation (Section Limitations). Age-standardized regional comparisons for 2024 (addressing whether regional differences in Table 1 reflect population age structure) could similarly not be computed because the publicly released regional tables report region × sex counts but not a region × age × sex cross-tabulation; this is also acknowledged as a limitation, and regional crude rates in Table 1 should accordingly be interpreted with caution given the steep age gradients shown in Section 3.2.

### 2.6. Forecast Modeling and Validation

Forecasts for 2025–2028 used binomial logit generalized linear models (GLMs) for abnormal and normal counts (the term “nonabnormal” used in an earlier draft has been replaced throughout) with calendar year as a linear predictor [14,15]. Because the analysis used aggregate annual prevalence data with only 13 (eGFR) or 15 (other indicators) annual observations, and because treating large aggregate counts as binomial trials substantially understates true forecast uncertainty—the effective independent unit for estimating the temporal trend is the year, not the individual examinee—three additional analyses were conducted to characterize and communicate this uncertainty more realistically. First, temporal hold-out validation was performed for each indicator by refitting the binomial logit GLM using only 2012–2020 data and comparing predicted values for 2021–2024 with the corresponding observed rates. This validation showed close agreement for eGFR, serum creatinine, and dipstick proteinuria (mean absolute error 0.05–0.14 percentage points across the four held-out years) but substantially poorer agreement for triglycerides and the anemia proxy (mean absolute error 1.5–1.7 percentage points), indicating that the linear-trend model does not adequately capture recent trajectory changes for these two indicators. Second, a sensitivity analysis refit each model using only the most recent seven years (2018–2024); for eGFR, creatinine, and proteinuria, the recent-window trend coefficient retained the same sign and a broadly similar magnitude as the full-period (2012–2024) model, but for triglycerides and the anemia proxy the recent-window model produced a materially different, and for anemia an opposite-signed, trend coefficient. On the basis of these two diagnostics, the eGFR, creatinine, and proteinuria forecasts are presented as the primary quantitative forecasts, whereas the triglyceride and anemia-proxy forecasts are presented as exploratory scenario projections only, and inferential claims regarding their precision are avoided (Section 3.1 and Section 4). Third, 95% prediction intervals for the 2025–2028 forecasts were derived using a residual-based parametric bootstrap (5000 resamples) that combined (i) sampling uncertainty in the fitted trend and intercept coefficients from their estimated standard errors and (ii) resampling of the empirical in-sample residuals (observed–fitted annual prevalence, 2012–2024) to reflect year-to-year variability not captured by a simple linear trend; the resulting bootstrap prediction intervals are 10–100-fold wider than the nominal binomial confidence intervals reported in an earlier draft and are reported as the primary measure of forecast uncertainty in Table 2. Denominator counts for forecast years were extrapolated using log-linear regression on annual total examined counts; this extrapolation was retained because the recombination of forecast prevalence with a forecast denominator was used to report an approximate forecast abnormal count alongside the forecast rate (Table 2), and is not required if only the forecast prevalence itself is of interest. To maintain comparability across endpoints, primary forecast models used 2012–2024 because eGFR data began in 2012; a supplementary model using the full 2010–2024 series for the four indicators with earlier data produced trend coefficients consistent in sign and magnitude with the 2012–2024 models (not tabulated). Model fit was additionally summarized using Akaike Information Criterion (AIC) values (Table 3).

**Table 2:** National observed 2024 rates and model-based 2025–2028 forecasts.

Indicator	Source Years	Modeled Years	2024 Observed Rate, %	2024 Abnormal/Total	2025 Predicted Rate, % (95% PI)	2026 Predicted Rate, % (95% PI)	2027 Predicted Rate, % (95% PI)	2028 Predicted Rate, % (95% PI)
eGFR < 60 mL/min/1.73 m^2^	2012–2024	2012–2024	3.43	601,125/17,507,116	3.20 (3.03–3.41)	3.15 (2.98–3.36)	3.11 (2.93–3.31)	3.06 (2.88–3.27)
Serum creatinine ≥ 1.5 mg/dL	2010–2024	2012–2024	0.83	144,706/17,516,863	0.75 (0.69–0.83)	0.74 (0.69–0.82)	0.73 (0.68–0.81)	0.73 (0.67–0.80)
Dipstick proteinuria ≥ 1+	2010–2024	2012–2024	3.71	646,011/17,423,959	3.98 (3.83–4.16)	4.19 (4.04–4.37)	4.41 (4.26–4.59)	4.64 (4.50–4.83)
Triglycerides ≥ 150 mg/dL	2010–2024	2012–2024	26.67	1,603,822/6,013,843	27.51 (26.55–29.37)	27.43 (26.47–29.29)	27.35 (26.39–29.21)	27.27 (26.31–29.14)
Sex-specific anemia proxy	2010–2024	2012–2024	7.04	1,232,802/17,516,433	6.19 (5.22–7.22)	5.97 (5.00–7.00)	5.76 (4.79–6.79)	5.56 (4.58–6.58)

Note: Forecasts used binomial logit GLMs with calendar year as the linear predictor on the logit scale. Intervals are 95% bootstrap prediction intervals (5000 resamples) combining trend-coefficient sampling uncertainty with resampled in-sample residual variability (Section 2.6); they are substantially wider than nominal binomial confidence intervals but still assume continuation of the historical linear trend and do not capture structural changes in screening participation, policy, or measurement. Triglyceride and anemia-proxy forecasts showed poor hold-out validation performance (Table 4) and should be interpreted as exploratory scenario projections rather than precise quantitative predictions.

**Table 3:** Forecast model trend coefficients and 2028 rates.

Indicator	Trend Coefficient (Logit/Year)	Trend *p* Value	2028 Predicted Rate, %
Serum creatinine ≥ 1.5 mg/dL	−0.0108	<0.001	0.73
eGFR < 60 mL/min/1.73 m^2^	−0.0161	<0.001	3.06
Sex-specific anemia proxy	−0.0383	<0.001	5.56
Dipstick proteinuria ≥ 1+	0.0538	<0.001	4.64
Triglycerides ≥ 150 mg/dL	−0.0040	<0.001	27.27

Note: Trend coefficients are on the logit scale. *p* values reflect aggregate counts and large sample sizes.

**Table 4:** Age-standardized versus crude national annual prevalence, selected years.

Indicator	2012 Crude, %	2012 Standardized, %	2018 Crude, %	2018 Standardized, %	2024 Crude, %	2024 Standardized, %
eGFR < 60 mL/min/1.73 m^2^	4.13	5.20	3.61	3.96	3.43	3.43
Serum creatinine ≥ 1.5 mg/dL	0.93	1.10	0.80	0.87	0.83	0.83
Dipstick proteinuria ≥ 1+	2.20	2.34	2.62	2.67	3.71	3.71
Triglycerides ≥ 150 mg/dL	28.61	29.51	29.93	29.63	26.67	26.67

Note: Standardized rates were directly age-standardized to the 2024 national screening population’s age distribution (16 five-year age bands, sex-combined); 2024 crude and standardized rates are therefore identical by construction. The age-standardized eGFR < 60 trend declined more steeply than the crude trend (5.20% → 3.43%, vs. 4.13% → 3.43% crude), and the standardized proteinuria trend rose in parallel with the crude trend (2.34% → 3.71%, vs. 2.20% → 3.71% crude), indicating that neither signal is an artifact of the aging screened population. An age-stratified hemoglobin table was not available; the anemia proxy could not be age-standardized.

### 2.7. Ethics Statement

This study used publicly available aggregate data without individual-level identifiers, obtained from Korean National Health Insurance Service health-screening statistics distributed through KOSIS. In accordance with institutional guidelines and national regulations, this study was granted exemption from Institutional Review Board review, and the requirement for informed consent was waived because no identifiable personal information was included in the dataset [16] (IRB No. 202601-SB-014-01; approved 20 January 2026).

## 3. Results

### 3.1. National Trends and Forecasts

**Figure 1:**
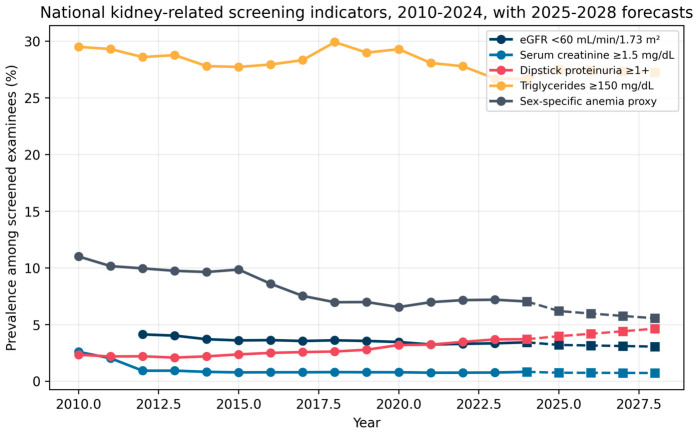
National observed trends and 2025–2028 model-based forecasts for kidney-related screening indicators. Solid lines represent observed data; dashed segments represent model-based forecasts from binomial logit GLMs [11,12].

**Figure 2:**
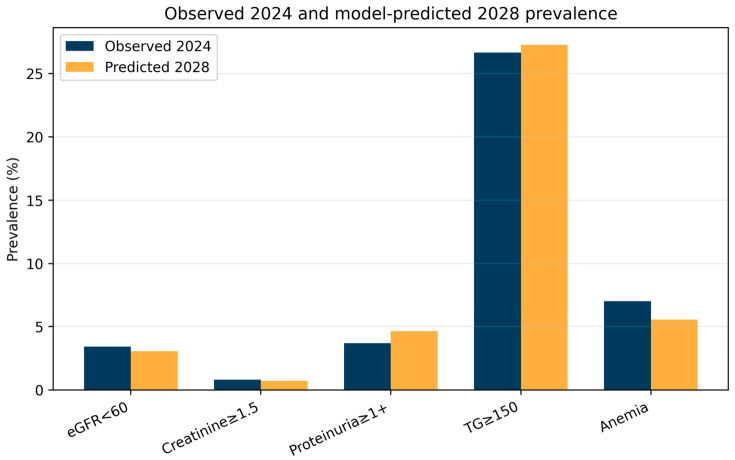
Observed 2024 and predicted 2028 abnormal screening prevalence across all endpoints.

**Figure 3:**
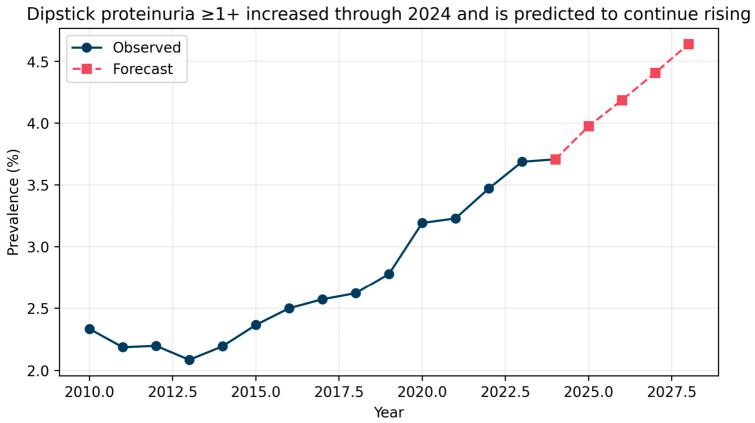
Dipstick proteinuria ≥ 1+ trend and forecast, 2010–2028.

**Figure 4:**
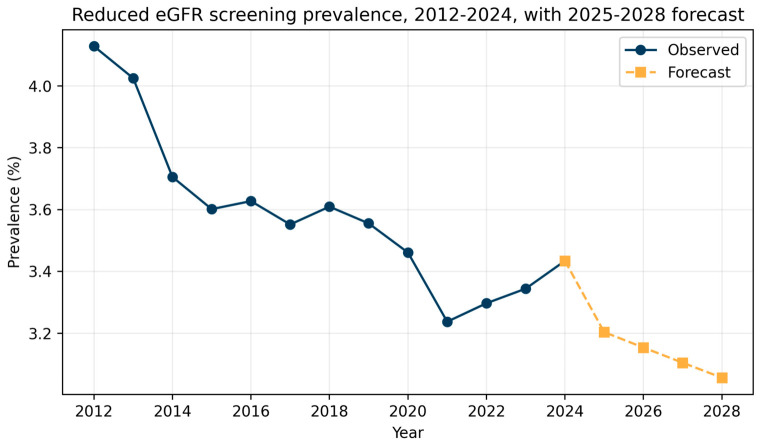
Reduced eGFR screening prevalence trend and forecast, 2012–2028.

Observed national trends and 2025–2028 forecasts are summarized in Table 2 and Table 3, and illustrated in Figure 1, Figure 2, Figure 3 and Figure 4. Dipstick proteinuria ≥ 1+ increased from 2.20% in 2012 to 3.71% in 2024 and was forecast to increase further to 4.64% (95% bootstrap prediction interval [PI], 4.50–4.83%) by 2028 (Table 2). The positive trend coefficient for proteinuria was statistically significant (*p* < 0.001; Table 3), and age-standardized proteinuria prevalence (Table 4) rose in parallel with the crude trend (2.34% in 2012 to 3.71% in 2024, using the 2024 age distribution as the reference population), confirming that the rising signal is not an artifact of the aging screened population. In contrast, eGFR < 60 declined from 4.13% in 2012 to 3.43% in 2024 and was forecast to be 3.06% (95% PI, 2.88–3.27%) in 2028; the age-standardized eGFR < 60 trend declined even more steeply than the crude trend (5.20% in 2012 to 3.43% in 2024, Table 4), indicating that the apparent crude decline is not explained by, and is if anything partly masked by, the aging of the screened population over this period. These aggregate-level abnormal screening prevalence figures reflect screening-based kidney risk indicators and are proxy indicators rather than confirmed CKD cases. Elevated serum creatinine also had a statistically significant downward model-based trend that was robust to age standardization and to the choice of modeling window (Table 4). Temporal hold-out validation (fitting each model through 2020 and predicting 2021–2024; Table 5) showed close agreement between predicted and observed values for eGFR, creatinine, and proteinuria (mean absolute error 0.05–0.14 percentage points), supporting the reliability of these three forecasts (Table 5). By contrast, hold-out validation and a recent-window (2018–2024) sensitivity analysis both indicated poor recent model fit for triglycerides and the anemia proxy (mean absolute error 1.5–1.7 percentage points; sign of the trend coefficient for the anemia proxy reversed in the recent-window model; Table 5). The triglyceride and anemia-proxy forecasts in Table 2 are therefore presented as exploratory scenario projections rather than as reliable quantitative predictions, and the apparent downward model-based trends for these two indicators (Table 3) should be interpreted with substantial caution. Sex-stratified national trends and forecasts (Table 6) showed that dipstick proteinuria increased in both sexes but more steeply in men (4.09% in 2024 rising to a projected 5.22% in 2028) than in women (3.29% rising to 4.04%), while the anemia proxy was consistently far more prevalent in women (10.79% in 2024) than in men (3.56%), consistent with menstrual and reproductive-age iron losses in the female subgroup contributing disproportionately to this indicator.

### 3.2. Age-Specific Findings

**Figure 5:**
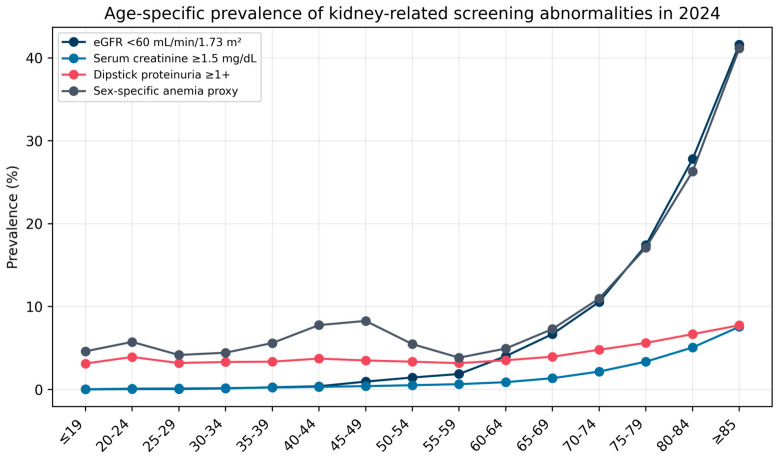
Age-specific prevalence of kidney-related screening abnormalities in 2024, for eGFR < 60 mL/min/1.73 m^2^, serum creatinine ≥ 1.5 mg/dL, dipstick proteinuria ≥1+, and the sex-specific anemia proxy. Triglycerides are not shown because triglyceride prevalence (24–37% across age groups) is on a substantially different scale from the other four endpoints (0–42%) and its age pattern (highest in mid-adulthood) differs qualitatively from the monotonic age gradients of the other indicators; the triglyceride age distribution is reported separately in Table 7.

Kidney function abnormalities were strongly age-concentrated (Table 7; Figure 5). In 2024, the highest eGFR < 60 prevalence occurred among persons aged ≥ 85 years (41.61%), followed by those aged 80–84 years (27.80%) and 75–79 years (17.41%). The anemia proxy showed a similar age gradient, reaching 41.14% among persons aged ≥ 85 years. These patterns confirm that abnormal screening prevalence is disproportionately concentrated in older age groups and that these are proxy indicators rather than confirmed diagnoses [6,8].

### 3.3. Regional Findings and Correlations

**Figure 6:**
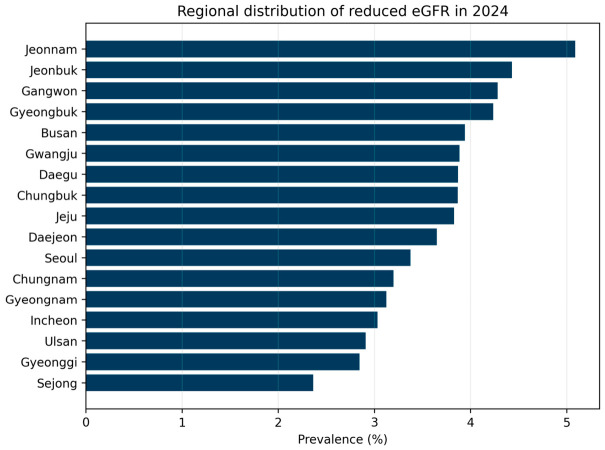
Regional distribution of reduced eGFR (<60 mL/min/1.73 m^2^) across 17 Korean administrative regions in 2024.

**Figure 7:**
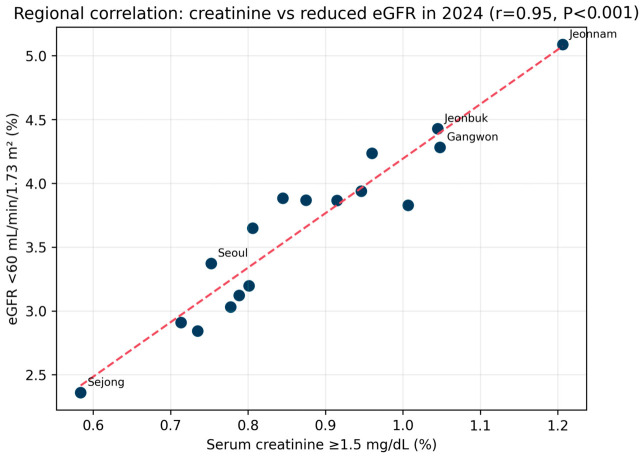
Regional correlation between elevated serum creatinine (≥1.5 mg/dL) and reduced eGFR (<60 mL/min/1.73 m^2^) in 2024 (Pearson r = 0.954, *p* < 0.001). Each circle represents one of the 17 administrative regions; the red dashed line is the ordinary least-squares linear fit.

Regional crude prevalence varied across 17 administrative regions (Table 1; Figure 6 and Figure 7). In 2024, Jeonnam had the highest crude eGFR < 60 prevalence (5.09%), while Sejong had the lowest (2.36%); because these are crude, not age-standardized, regional rates and Section 3.2 shows steep age gradients in eGFR < 60 and the anemia proxy, regional differences in Table 1 may substantially reflect differences in regional population age structure rather than differences in underlying kidney-risk exposure, and should not be interpreted as evidence of higher regional kidney risk; region-by-age cross-tabulated data needed to compute age-standardized regional rates were not available in the public KOSIS release (Section 2.5), and this is an acknowledged limitation. Region-level eGFR < 60 was strongly correlated with elevated serum creatinine (Pearson r = 0.954, 95% CI 0.876–0.983, *p* < 0.001; Spearman rho = 0.934, *p* < 0.001; Table 8; Figure 7); because eGFR is calculated from serum creatinine together with age and sex, these two indicators are mathematically coupled rather than independent measurements, and this strong correlation is expected by construction and is not presented as an independent validation of the kidney function signal. eGFR < 60 was also significantly correlated with the anemia proxy (Pearson r = 0.611, 95% CI 0.184–0.845, *p* = 0.009), a plausible but genuinely independent ecological association given that anemia is not derived from creatinine or eGFR. Correlations with proteinuria and triglycerides were not statistically significant. Because six pairwise regional correlations were tested (Table 8), and because estimates are based on only 17 regions and may be sensitive to individual high- or low-prevalence regions, these results are interpreted qualitatively and with attention to multiple comparisons rather than as a family of independent confirmatory tests; formal leave-one-region-out influence analysis was not performed and is noted as a direction for future work. These regional correlations are ecological associations and represent patterns in screening-based kidney risk indicators across administrative units [17]; interpretations involving occupational exposure, healthcare access, or comorbidity burden are speculative because these variables were not measured in this dataset and are not advanced as explanations here [14].

## 4. Discussion

This national aggregate-data analysis identified a statistically significant, age-standardization-robust rising signal in dipstick proteinuria through 2024, with a continued model-based increase projected through 2028 (Table 2 and Table 4). Several non-mutually exclusive explanations for this rising proteinuria signal should be considered. First, increasing prevalence of diabetes, hypertension, and obesity in the Korean population over this period could plausibly increase glomerular protein leakage independent of population aging, since the age-standardized trend persisted after accounting for the changing age structure of the screened population. Second, changes in screening participation—for example, if examinees with known cardiometabolic risk factors became more likely to attend screening over time—could inflate the apparent trend without a true rise in population-level risk; this cannot be excluded with the available aggregate data. Third, changes in dipstick reagent strips, urine collection procedures, or laboratory reporting practices over a 15-year period could shift the apparent positive rate independent of true proteinuria prevalence; this information is not reported in the public KOSIS tables and is an important avenue for verification by the data custodian. Fourth, increased clinical and screening-program awareness of albuminuria/proteinuria as a CKD risk marker could have increased test uptake or lowered the threshold for recording trace-to-1+ results as abnormal. Fifth, broader use of renin–angiotensin system inhibitors and sodium–glucose cotransporter-2 inhibitors, which can each independently lower measured proteinuria, argue against a medication-driven explanation for a rising signal, making a true increase in underlying risk-factor burden a more parsimonious explanation, though this cannot be confirmed without individual-level clinical data. This finding, taken together, supports policies that improve repeat urine testing, urine albumin-to-creatinine ratio confirmation when clinically appropriate, blood pressure and diabetes management, and follow-up pathways for persistent abnormalities [6,13,18,19], while acknowledging that this study did not examine confirmatory testing, referral, treatment, or clinical outcomes, and that the proteinuria trend should not, by itself, be used to infer a change in confirmed CKD incidence.

Reduced eGFR did not show a rising crude or age-standardized national trend; if anything, the age-standardized decline was steeper than the crude decline (Table 4), arguing against the simplest possible artifact (that population aging alone explains the apparent improvement). Several alternative, non-mutually exclusive explanations merit discussion rather than accepting the decline at face value as a true improvement in population kidney function. First, a mortality-selection (survivor) effect is plausible: if individuals with the most severely reduced eGFR are disproportionately likely to die, be hospitalized, or otherwise not re-present for routine screening as they age within the study period, the surviving screened population in later years could show a lower observed eGFR < 60 prevalence even if true age-specific risks in the underlying population were unchanged—a hypothesis consistent with the very steep age gradient in eGFR < 60 shown in Section 3.2 and one that the present ecological, repeated cross-sectional data cannot directly test. Second, if the eGFR-estimating equation, creatinine assay standardization (for example, the adoption of isotope-dilution mass spectrometry-traceable creatinine calibration), or reporting categories used by NHIS changed at any point between 2012 and 2024 (Section 2.3), an apparent temporal change in reduced-eGFR prevalence could result that does not reflect a true change in kidney function; because this information is not disclosed in the public aggregate tables, this possibility cannot be excluded and is highlighted as a priority for verification with NHIS in future collaborative work. Third, secular improvements in the management of diabetes, hypertension, and cardiovascular risk factors among screened adults could plausibly produce a true decline in reduced-eGFR prevalence at a given age, consistent with international trends in some CKD risk factors over the same period [20]. The regional concordance between reduced eGFR and elevated serum creatinine (Table 8) reflects the mathematical dependence of the eGFR equation on serum creatinine, age, and sex rather than an independent validation of the kidney function signal, and should not be interpreted as internal consistency evidence; this correlation is expected by construction. This differs from the correlation coefficients described in some clinical cohort studies of glomerulopathy, which are typically well below 0.6 because those cohorts examine within-patient longitudinal change under variable non-renal influences on creatinine (for example, muscle mass, hydration, and dietary protein intake); the very high ecological (region-level, cross-sectional) correlation reported here reflects aggregation over large populations rather than a tight individual-level physiological relationship, and the two should not be numerically compared. By contrast, the weaker but still statistically significant regional correlation between eGFR < 60 and the anemia proxy (Pearson r = 0.611) is a genuinely independent ecological association, consistent with reduced erythropoietin production and iron-handling changes accompanying reduced kidney function, whereas the nonsignificant correlations between eGFR and proteinuria suggest that reduced kidney function and proteinuria may capture overlapping but not identical screening-risk profiles at the ecological level [1,2], consistent with prior reports that eGFR and proteinuria capture partially distinct dimensions of CKD pathophysiology [6,7].

The overall national prevalence of reduced eGFR observed in this screened population (approximately 3.4–4.1% across 2012–2024) is lower than some global CKD prevalence estimates of approximately 9–10% [3,4]. Several factors specific to the Korean screening context, rather than an implausibly low true burden of kidney dysfunction, likely explain this difference. First, this study’s operational threshold (eGFR < 60 mL/min/1.73 m^2^ on a single screening measurement) is a more restrictive single-occasion definition than the persistence-based KDIGO CKD definition used in many prevalence surveys, which additionally captures albuminuric CKD with preserved eGFR and requires only mild-to-moderate reductions sustained over three months; a single-occasion, more severe threshold is expected to yield a lower estimated prevalence than a persistence-inclusive definition applied to the general population. Second, the NHIS screening population is not a random sample of the general Korean population: it comprises adults who actively attend a biennial employment- or insurance-linked screening program, which may under-represent the frailest, most severely ill, or institutionalized individuals who are simultaneously most likely to have severely reduced kidney function but least likely to attend ambulatory screening—a form of participation-related selection that would bias observed prevalence downward relative to true population prevalence. Third, South Korea’s near-universal National Health Insurance coverage and structured, employer- and government-linked biennial screening program provide comparatively early and systematic detection and management of diabetes and hypertension, the two leading CKD risk factors, which may generate a genuinely lower burden of advanced kidney dysfunction in the screened, insured population relative to global comparator populations with less comprehensive screening infrastructure; this is a plausible contributor but cannot be directly confirmed with the aggregate data used here. These explanations are not mutually exclusive, and disentangling them would require linked screening-participation and clinical outcome data beyond the scope of this ecological analysis.

Reduced eGFR and dipstick proteinuria are the two indicators directly relevant to CKD risk classification under KDIGO criteria and were treated as the primary screening endpoints in this analysis (Section 2.3). Elevated triglycerides and the anemia proxy were included as associated metabolic and hematological screening indicators, not as kidney-screening endpoints in the same sense as eGFR and proteinuria: reduced kidney function is a well-established, bidirectional correlate of both dyslipidemia (through altered lipoprotein metabolism and lipase activity) and anemia (through reduced erythropoietin production), such that population-level co-monitoring of these indicators alongside eGFR and proteinuria can help characterize the broader cardiometabolic and hematological risk profile that frequently accompanies reduced kidney function, even though neither indicator is diagnostic of CKD on its own. This conceptual framework, and the associated terminological distinction, is now stated explicitly in Section 2.3 and is reiterated here as a caution against over-interpreting the triglyceride and anemia-proxy trends as kidney-specific findings.

With respect to the proteinuria screening methodology, dipstick proteinuria (used here) and albuminuria (measured by urine albumin-to-creatinine ratio) capture overlapping but non-identical aspects of glomerular injury: dipstick tests are more sensitive to gross proteinuria including non-albumin proteins and are commonly used as an inexpensive first-line population screening tool, whereas quantitative albuminuria testing is more sensitive for early, predominantly glomerular injury and is specifically incorporated into the KDIGO CKD risk-classification heat-map [6]. Because dipstick proteinuria can reflect transient, orthostatic, or non-renal causes of proteinuria in addition to persistent glomerular damage, a positive dipstick screening result in this dataset should be regarded as a trigger for confirmatory quantitative albuminuria or repeat testing rather than as equivalent evidence of irreversible glomerular injury; this is consistent with, and reinforces rather than contradicts, this study’s overall framing of all screening endpoints as proxy indicators requiring confirmatory testing rather than confirmed CKD diagnoses.

The age-stratified findings underscore that kidney function-related screening indicators are disproportionately concentrated in older adults, consistent with the known age-related decline in GFR and the global epidemiology of CKD in elderly populations [8,9]. The crude regional variation in eGFR < 60, with Jeonnam and other predominantly rural, older-population provinces showing higher prevalence than metropolitan areas such as Seoul, Gyeonggi, and Sejong, is compatible with regional differences in population age structure (Section 3.2 and Section 3.3); rural provinces in Korea have older population pyramids than the metropolitan capital region, and this is considered the most parsimonious explanation for the observed pattern pending age-standardized regional data. Region-specific environmental exposures potentially relevant to kidney risk that could be examined in future linked studies include heavy-metal and agrochemical exposure in agricultural provinces, water hardness and mineral content, and industrial or occupational exposures near manufacturing centers, but none of these were measured in this dataset, and the present findings should not be used to draw conclusions about specific environmental determinants without further, purpose-designed investigation combining regional exposure data with age-standardized health outcomes.

The forecasts should be interpreted as model-based projections of screening prevalence proxies, not as predicted CKD diagnoses, and their reliability differs materially by indicator (Section 2.6, Table 5). The eGFR, serum creatinine, and dipstick proteinuria forecasts showed good temporal hold-out validation performance and directionally stable trend estimates across modeling windows, supporting their use as the primary quantitative forecasts in this study. The triglyceride and anemia-proxy forecasts, by contrast, showed poor hold-out fit and trend instability across modeling windows and are presented as exploratory scenario projections only; in particular, the reversal in sign of the anemia-proxy trend coefficient between the full-period and recent-window models indicates that the modest downward anemia-proxy trend reported in Table 3 should not be treated as an established or continuing trajectory. Changes in screening participation, laboratory practices, population structure, policy, or clinical follow-up may alter observed future rates for all indicators [11,12]. Bootstrap prediction intervals reported in Table 2 (Section 2.6) are substantially wider than the nominal binomial confidence intervals used in an earlier draft of this analysis and better reflect year-to-year variability, but they still assume continuation of a linear logit-scale trend and do not capture structural discontinuities such as changes in screening policy or the eGFR-estimating equation.

### Limitations

This study has several limitations. First, the analysis used aggregate screening tables rather than individual-level longitudinal records; therefore, patient-level incidence estimation, covariate adjustment, comorbidity assessment, medication effects, de-duplication of repeat examinees across years, and causal inference were not possible [14,15]. Second, eGFR < 60 and dipstick proteinuria ≥1+ are screening-based kidney risk indicators and proxy indicators rather than confirmed CKD diagnoses; in particular, persistence for at least three months could not be assessed in this dataset [6,7]. Third, regional correlations are ecological, based on only 17 regions, not age-standardized because region × age cross-tabulated data were unavailable, and cannot be generalized to individual-level associations; regional rankings (Table 1) should therefore be interpreted cautiously pending age-standardized regional analysis. Fourth, 2025–2028 forecasts assume the continuation of historical linear trends and, for triglycerides and the anemia proxy specifically, showed poor temporal hold-out validation performance and trend instability across modeling windows (Section 2.6, Table 5); these two forecasts are presented as exploratory only. Fifth, the specific eGFR-estimating equation used by NHIS, whether it changed between 2012 and 2024, and whether serum creatinine assay calibration was standardized over this period could not be verified from the publicly released aggregate tables; any such change could produce an apparent temporal change in reduced-eGFR prevalence unrelated to true kidney function change (Section 2.3). Sixth, an age-stratified hemoglobin table was not available, so the anemia proxy could not be age-standardized, and age × region cross-tabulated data were not available for any indicator. Seventh, screening eligibility, participation rates, and the specific screening programs or examination types combined within each KOSIS table in 2010–2024 could not be fully verified from the publicly released tables; changes in screening participation composition over time cannot be excluded as a contributor to the observed national trends. Eighth, six pairwise regional correlations were tested without formal multiple-testing correction or leave-one-region-out influence analysis; results should be interpreted qualitatively. Ninth, these data do not include renal cancer incidence, renal mortality, or tumor-registry endpoints.

## 5. Conclusions

Using NHIS/KOSIS aggregate health-screening data, this study found a significant, age-standardization-robust increase in dipstick proteinuria screening prevalence, a crude and age-standardized decline in reduced eGFR that was not explained by population aging, strong age concentration of reduced eGFR and anemia proxy indicators, and a regional correlation between reduced eGFR and elevated serum creatinine that reflects their mathematical dependence rather than independent validation. Model-based forecasts, validated by temporal hold-out testing and cross-checked against a recent-window sensitivity analysis, indicate that the proteinuria, eGFR, and serum creatinine trends are reasonably reliable through 2028, whereas the triglyceride and anemia-proxy forecasts showed weaker validation performance and are presented as exploratory only. These findings support confirmatory testing after abnormal screening results and targeted kidney-risk follow-up programs, particularly in older age groups and, pending age-standardized confirmation, higher-prevalence regions. However, results should not be interpreted as confirmed CKD incidence or prevalence, as all endpoints represent screening-based kidney risk indicators and proxy indicators rather than adjudicated diagnoses [6,18].

## Figures and Tables

**Table 1:** Regional crude prevalence across screening endpoints in 2024.

Region	eGFR < 60, %	Creatinine ≥ 1.5, %	Proteinuria ≥ 1+, %	Triglycerides ≥ 150, %	Anemia Proxy, %
Jeonnam	5.09	1.21	3.22	28.50	8.58
Jeonbuk	4.43	1.04	3.62	28.14	8.57
Gangwon	4.28	1.05	3.81	27.37	7.04
Gyeongbuk	4.24	0.96	3.65	27.28	8.32
Busan	3.94	0.95	4.07	24.93	7.50
Gwangju	3.88	0.84	3.58	27.31	7.24
Daegu	3.87	0.87	3.42	25.72	8.22
Chungbuk	3.87	0.91	3.31	28.38	6.94
Jeju	3.83	1.01	4.41	24.40	7.04
Daejeon	3.65	0.81	3.32	27.15	7.17
Seoul	3.37	0.75	4.30	24.40	6.78
Chungnam	3.20	0.80	3.24	29.38	6.86
Gyeongnam	3.12	0.79	3.42	26.57	7.80
Incheon	3.03	0.78	3.90	28.05	6.77
Ulsan	2.91	0.71	2.70	26.90	6.65
Gyeonggi	2.84	0.73	3.66	27.19	6.18
Sejong	2.36	0.58	2.58	24.13	7.63

Note: Regional rates are crude and not age-standardized.

**Table 5:** Forecast model diagnostics, hold-out validation, and sensitivity to modeling window.

Indicator	AIC (2012–2024 Model)	Hold-Out MAE, pp (2021–2024)	Recent-Window (2018–2024) Trend Coefficient	Trend Sign Consistent with Full Model?
eGFR < 60 mL/min/1.73 m^2^	7491.3	0.12	−0.0116 (*p* < 0.001)	Yes
Serum creatinine ≥ 1.5 mg/dL	3830.8	0.05	−0.0007 (*p* = 0.215)	Yes (attenuated)
Dipstick proteinuria ≥ 1+	6292.1	0.14	0.0610 (*p* < 0.001)	Yes
Triglycerides ≥ 150 mg/dL	27,209.7	1.69	−0.0279 (*p* < 0.001)	Yes (larger magnitude)
Sex-specific anemia proxy	101,836.6	1.47	0.0067 (*p* < 0.001)	No (sign reversed)

Note: AIC values are from the primary 2012–2024 binomial logit GLM (Table 3). Hold-out mean absolute error (MAE) compares model predictions (fitted using only 2012–2020 data) with observed 2021–2024 rates, in percentage points (pps). The recent-window model refits the same model specification using only 2018–2024 data. eGFR, creatinine, and proteinuria forecasts are considered reasonably robust to model specification and are reported as primary quantitative forecasts in Table 2. Triglyceride and anemia-proxy forecasts showed poor hold-out fit and/or trend instability and are reported as exploratory scenario projections only.

**Table 6:** Sex-stratified national observed 2024 rates and 2025–2028 forecasts.

Indicator	Sex	2024 Observed Rate, %	2025 Predicted Rate, %	2026 Predicted Rate, %	2027 Predicted Rate, %	2028 Predicted Rate, %
Serum creatinine ≥ 1.5 mg/dL	Men	1.29	1.21	1.21	1.21	1.21
	Women	0.32	0.27	0.26	0.25	0.24
eGFR < 60 mL/min/1.73 m^2^	Men	3.63	3.43	3.46	3.49	3.52
	Women	3.22	3.00	2.88	2.77	2.66
Sex-specific anemia proxy	Men	3.56	3.09	3.02	2.94	2.87
	Women	10.79	9.37	8.95	8.55	8.17
Dipstick proteinuria ≥ 1+	Men	4.09	4.43	4.68	4.94	5.22
	Women	3.29	3.50	3.67	3.85	4.04
Triglycerides ≥ 150 mg/dL	Men	33.72	34.47	34.28	34.08	33.88
	Women	18.06	19.10	19.15	19.20	19.25

Note: Sex-stratified forecasts use the same binomial logit GLM specification as Table 2, fitted separately by sex. Men consistently showed higher triglyceride and eGFR < 60 prevalence, whereas women showed markedly higher anemia-proxy prevalence at all time points, consistent with menstrual-related iron loss in reproductive-age women; this sex difference narrows the interpretability of the sex-combined anemia-proxy trend reported in Table 2 and Table 3.

**Table 7:** Highest-risk age groups for selected screening endpoints in 2024.

Indicator	Age Group	Total Examined	Abnormal Count	Rate, %
Serum creatinine ≥ 1.5 mg/dL	≥85	107,821	8162	7.57
Serum creatinine ≥ 1.5 mg/dL	80–84	356,867	18,106	5.07
Serum creatinine ≥ 1.5 mg/dL	75–79	475,565	16,007	3.37
Serum creatinine ≥ 1.5 mg/dL	70–74	1,083,975	23,543	2.17
Serum creatinine ≥ 1.5 mg/dL	65–69	1,284,661	17,568	1.37
eGFR < 60 mL/min/1.73 m^2^	≥85	107,722	44,821	41.61
eGFR < 60 mL/min/1.73 m^2^	80–84	356,547	99,109	27.80
eGFR < 60 mL/min/1.73 m^2^	75–79	475,221	82,733	17.41
eGFR < 60 mL/min/1.73 m^2^	70–74	1,083,169	114,526	10.57
eGFR < 60 mL/min/1.73 m^2^	65–69	1,283,600	85,865	6.69
Sex-specific anemia proxy	≥85	107,816	44,352	41.14
Sex-specific anemia proxy	80–84	356,857	93,882	26.31
Sex-specific anemia proxy	75–79	475,556	81,370	17.11
Sex-specific anemia proxy	70–74	1,083,945	119,117	10.99
Sex-specific anemia proxy	45–49	1,608,588	133,132	8.28
Dipstick proteinuria ≥ 1+	≥85	100,423	7784	7.75
Dipstick proteinuria ≥ 1+	80–84	349,780	23,385	6.69
Dipstick proteinuria ≥ 1+	75–79	470,782	26,472	5.62
Dipstick proteinuria ≥ 1+	70–74	1,076,720	51,663	4.80
Dipstick proteinuria ≥ 1+	65–69	1,279,132	50,658	3.96
Triglycerides ≥ 150 mg/dL	35–39	213,470	79,402	37.20
Triglycerides ≥ 150 mg/dL	30–34	233,705	71,893	30.76
Triglycerides ≥ 150 mg/dL	45–49	552,937	165,314	29.90
Triglycerides ≥ 150 mg/dL	50–54	592,682	174,551	29.45
Triglycerides ≥ 150 mg/dL	40–44	1,038,866	294,724	28.37

Note: Rows show the top five age groups within each endpoint by 2024 observed prevalence.

**Table 8:** Regional correlations among screening indicators in 2024.

Comparison	n Regions	Pearson r (*p* Value)	Spearman rho (*p* Value)
eGFR vs. creatinine	17	0.954 (<0.001)	0.934 (<0.001)
eGFR vs. proteinuria	17	0.302 (0.238)	0.189 (0.468)
eGFR vs. triglycerides	17	0.306 (0.232)	0.385 (0.127)
eGFR vs. anemia	17	0.611 (0.009)	0.635 (0.006)
Proteinuria vs. triglycerides	17	−0.258 (0.318)	−0.250 (0.333)
Creatinine vs. anemia	17	0.537 (0.026)	0.574 (0.016)

Note: Correlations are ecological region-level associations and do not imply individual-level causal relationships.

## Data Availability

The analysis was based on aggregate health-screening statistics publicly available from the Korean National Health Insurance Service (https://www.nhis.or.kr (accessed on 15 May 2026)) [12] and the Korean Statistical Information Service (https://kosis.kr (accessed on 15 May 2026)) [11]. The specific KOSIS aggregate tables retrieved were the National Health Insurance Service health-screening result distributions by region and by age group for estimated glomerular filtration rate, serum creatinine, urine protein (dipstick), triglycerides, and hemoglobin (KOSIS series accessed 15 May 2026; table titles: “Regional Distribution of Estimated Glomerular Filtration Rate” (Sido-byeol Sinsagucheveogwayul Bunpohyeonhwang), “Age-Specific Distribution of Estimated Glomerular Filtration Rate” (Yeonryeong-byeol Sinsagucheveogwayul Bunpohyeonhwang), “Regional Distribution of Serum Creatinine” (Sido-byeol Hyeolcheongcreatinine Bunpohyeonhwang), “Age-Specific Distribution of Serum Creatinine” (Yeonryeong-byeol Hyeolcheongcreatinine Bunpohyeonhwang), “Regional Distribution of Urine Protein” (Sido-byeol Yodanbaek Bunpohyeonhwang), “Age-Specific Distribution of Urine Protein” (Yeonryeong-byeol Yodanbaek Bunpohyeonhwang), “Regional Distribution of Triglycerides” (Sido-byeol Triglyceride Bunpohyeonhwang), and “Age-Specific Distribution of Triglycerides” (Yeonryeong-byeol Triglyceride Bunpohyeonhwang)). The derived analytical datasets (annual national counts, age-standardized rates, regional rates, and forecast outputs) and the R analysis code used to generate all statistics, figures, and tables in this manuscript are available from the corresponding authors upon reasonable request. Individual-level NHIS raw data were not used in this study.
